# Spatio-Temporal Dynamics of Field Cricket Calling Behaviour: Implications for Female Mate Search and Mate Choice

**DOI:** 10.1371/journal.pone.0165807

**Published:** 2016-11-07

**Authors:** Diptarup Nandi, Rohini Balakrishnan

**Affiliations:** Centre for Ecological Sciences, Indian Institute of Science, Bangalore, Karnataka, India; Museum National d'Histoire Naturelle, FRANCE

## Abstract

Amount of calling activity (calling effort) is a strong determinant of male mating success in species such as orthopterans and anurans that use acoustic communication in the context of mating behaviour. While many studies in crickets have investigated the determinants of calling effort, patterns of variability in male calling effort in natural choruses remain largely unexplored. Within-individual variability in calling activity across multiple nights of calling can influence female mate search and mate choice strategies. Moreover, calling site fidelity across multiple nights of calling can also affect the female mate sampling strategy. We therefore investigated the spatio-temporal dynamics of acoustic signaling behaviour in a wild population of the field cricket species *Plebeiogryllus guttiventris*. We first studied the consistency of calling activity by quantifying variation in male calling effort across multiple nights of calling using repeatability analysis. Callers were inconsistent in their calling effort across nights and did not optimize nightly calling effort to increase their total number of nights spent calling. We also estimated calling site fidelity of males across multiple nights by quantifying movement of callers. Callers frequently changed their calling sites across calling nights with substantial displacement but without any significant directionality. Finally, we investigated trade-offs between within-night calling effort and energetically expensive calling song features such as call intensity and chirp rate. Calling effort was not correlated with any of the calling song features, suggesting that energetically expensive song features do not constrain male calling effort. The two key features of signaling behaviour, calling effort and call intensity, which determine the duration and spatial coverage of the sexual signal, are therefore uncorrelated and function independently.

## Introduction

Across several taxa from insects to anurans, birds and mammals, animals that produce acoustic signals aggregate spatially to form choruses [1–3]. A functional chorus requires acoustic interactions between its participants. In orthopteran and anuran choruses, individuals, usually of one sex (typically the male), use acoustic signals to attract conspecific mates over a long distance, leading to intra-specific sexual competition [1]. The amount of time spent calling in a chorus is known to positively influence an individual’s mating success in crickets and anurans [1,4]. However, calling behaviour is energetically expensive and may also attract predators and parasites [1,5]. Energetic demands of calling and predation risk thus constitute the major costs of acoustic signaling.

Variation in individual calling behaviour shapes the temporal dynamics of the chorus.

Variation in male calling behaviour can be divided into among-individual and within- individual differences in calling activity across multiple nights. A multitude of field studies in several species of anurans have elucidated the within- and among-individual differences in calling activity in terms of chorus tenures across multiple nights [6–8]. These studies have shown that chorus tenures tend to vary across anuran species, from short abbreviated chorusing to prolonged continuous chorusing, primarily due to different breeding phenologies [9–13]. In orthopterans, field studies on the chorusing behaviour in different natural populations of the field cricket species *Gryllus campestris L* have primarily investigated behaviours such as timing of calling, presence of non-callers and burrow displacements, which may indirectly influence the temporal dynamics of chorusing [14–17]. Despite demonstrating the importance of calling behaviour in male mating success [15], these field studies did not quantify variability in calling activity. Some studies under semi-natural conditions on other species of field crickets such as *Gryllus veletis*, *Gryllus pennsylvanicus* and *Gryllus integer* investigated the among-individual variation in daily calling activity and mate searching behaviour under experimentally manipulated density regimes [18,19]. These studies were able to demonstrate that calling effort was positively associated with male mating success at low population densities.

Variation in calling activity in crickets and katydids has been generally studied under controlled laboratory conditions to examine the causes of such variation. Several studies have shown a positive effect of nutrition on the amount of time spent calling (calling effort) in different species of field crickets [20–23] (but see [24]). Calling effort has also been shown to depend more heavily on immediate adult nutritional condition than on nymphal nutritional condition [21,25]. Nutritional dependence of calling effort suggests calling to be an energetically expensive behaviour. The high metabolic costs of calling estimated in several species of crickets and katydids further corroborate the idea that calling is energetically expensive [26–28]. Physical competition, on the other hand, was found to have no effect on calling effort in the field cricket species *Teleogryllus commodus* [29]. Despite an understanding of the potential causes of variability in calling effort among males, the emergent patterns of within- and among-individual variation in their natural habitat still remain unexplored. Thus the first objective of this study was to quantify the variation in calling activity of male crickets in natural choruses in terms of the among- and within-individual components of variance at two temporal scales: within and across nights.

For females to assess potential mates based on their calling song, callers need to be actively signaling at least during the assessment period. The temporal pattern of chorusing can thus affect a female’s mate search strategy [30]. Pair formation during mating requires conspecific males and females to find each other. Since females use the male calling song over long distances to localise potential mates, it is more likely that the males remain stationary while the female searches for mates. In absence of any signaling by the females, unlike in duetting species, incessant male movement can constrain the female’s ability to locate callers. In many species of true crickets, males generally remain stationary while calling within a night [31–33]. Since females may also use acoustic signals to discriminate between conspecific males, the time window of female mate search is likely to be constrained by a male’s calling site fidelity.

Many of the theoretical models of mate sampling strategies require a female to remember and revisit previously sampled males [34–39]. Such strategies will only be operationally feasible if males do not change their calling sites frequently i.e. have high calling site fidelity. If callers have high calling site fidelity only within nights and change calling site frequently across nights, then females will tend to sample males within a night rather than across nights. On the other hand, if callers maintain their calling sites over several nights, then females have a longer time window for their mate search. Therefore the second objective of this study was to quantify movement of callers across their nights of calling to estimate across-night calling site fidelity.

While calling effort determines the amount of sexual signaling, acoustic signals have multiple temporal and spectral features that can be energetically costly and important for mate attraction as well [1,27]. In field crickets and katydids, calling song chirp rate is energetically expensive and co-varies positively with the immediate nutritional condition of the male [20,23,26,28,29]. Calling song sound pressure level (SPL), which is a measure of its loudness, is also known to be energetically expensive [1]. Female crickets generally prefer energetically expensive calling song features such as higher SPLs [1,40] and faster chirp rates [24,41]. Thus, similar to calling effort, signal components such as SPL and chirp rate are energetically costly and also function as mate attraction cues. In such a scenario, optimization of energetic investment in sexual signaling may lead to trade-offs between the different signal features and calling effort [42].

Theoretical models attempting to explain the existence of multiple cues in sexual signaling lead to diverse expectations. The ‘multiple messages’ hypothesis states that different signal components convey messages about different qualities of a signaler, and predicts a lack of correlation between the multiple components [43–46]. The ‘back-up signal’ or ‘redundant signal’ hypothesis suggests that the multiple components of a signal reinforce the message about signaler quality, allowing greater accuracy in signal evaluation. This hypothesis predicts a positive correlation between the different signal components [43–46]. Empirical studies in orthopterans and anurans have investigated trade-offs between the different temporal features of acoustic signals [47,48]. In Texas field crickets, *Gryllus texensis*, trade-offs between bout duration, number of bouts and trilling amplitude were found only in males with higher calling effort across multiple nights under laboratory conditions [42]. However, such trade-offs between calling effort and amplitude were absent in males with low across-night calling effort in the same study. Field studies on trade-offs between temporal features such as chirp rate, SPL and calling effort, which are critical mate attraction cues, are however lacking, especially in crickets and katydids. Thus the third objective in this study was to investigate correlations between calling effort and calling song features such as SPL and chirp rate in the field.

The objectives of this study were addressed in a field cricket species, *Plebeiogryllus guttiventris* [49]. Males of this species spatially aggregate to form choruses and use their acoustic advertisement signals to attract conspecific females over long distances [32]. Unlike some field cricket species that use burrows, *P*. *guttiventris* males call from naturally occurring cracks in the field. Moreover, callers rarely change their calling site within a night leading to high within-night site fidelity [32]. The male calling song of *P*. *guttiventris* has been characterized [32]. Male calling song SPL has been shown to play a critical role in female phonotaxis in complex acoustic scenarios, with females preferring louder calls [40,50]. In *Plebeiogryllus guttiventris*, SPL was the only calling song feature that was correlated with male body size [51]. Calling song SPL and other song features such as chirp rate were found to be uncorrelated [51]. In this species, calling song SPL and chirp rate were both found to play an important role in mate attraction, with SPL being the more dominant feature compared to chirp rate [51].

Mate attraction by acoustic signaling can be considered along a two-dimensional spatio-temporal axis. Calling effort determines mate attraction along the temporal axis by affecting the duration of the signaling behaviour. SPL determines the spatial axis of mate attraction by affecting the spatial broadcast area of the mate attraction signal.

Calling effort and calling song SPL are critical aspects of signaling behaviour that could be under sexual selection via different operative mechanisms [30,52–54]. Thus, variation in calling effort and its correlation with other signal components provide the basis for exploring the role of sexual selection in the evolution and maintenance of acoustic communication.

Therefore, in this study, we had three primary objectives. The first objective was to investigate consistency of calling activity of individual callers across multiple nights. To address this objective we quantified variation in individual calling effort across multiple nights in a wild population of the field cricket species *Plebeiogryllus guttiventris*. The second objective was to investigate across-night movement patterns of callers to estimate calling site fidelity across multiple nights. The third objective was to investigate possible trade-offs between calling effort and other components of the acoustic signal.

## Materials and Methods

### Ethics statement

All behavioural data collection protocols used in this study adhered to the national guidelines for the ethical treatment of animals laid out by the National Biodiversity Authority (Government of India). The field cricket used in this study is a common species distributed throughout India in agricultural and suburban areas and is not listed as endangered. All fieldwork pertaining to this study was conducted with the full consent and permission of the owner of the privately-owned agricultural fields.

### Study system

The study was conducted on a wild population of the field cricket species *Plebeiogryllus guttiventris*, in agricultural fields near village Ullodu (13°38′48.81″ N, 77°42′45.23″ E) in the southern Indian state of Karnataka, in the breeding season between the months of January and April. *Plebeiogryllus guttiventris* is nocturnal and males are acoustically active from 1900 to 2200 hrs. Thus all sampling was carried out during this period.

### Sampling

An initial *ad libitum* sampling was conducted for eight nights to capture wild calling males and to delineate the boundaries of the sampling area for future observations. The captured males were marked on their pronotum with a unique 3-colour combinatorial code for individual identification, using a nontoxic paint marker (Edding 780, Edding, St Albans, U.K.) and subsequently released near their calling sites. The boundaries of the sampling area were set such that either no callers were found beyond this area or the habitat was not found to be conducive for calling (tilled land on one side). The sampling arena thus consisted of a core area (28 m × 17 m) where most of the calling activity was found and a buffer area (approximately 10 m width) surrounding the core.

The systematic sampling to quantify calling effort was started after a day’s break from the initial sampling and was continued for 31 consecutive nights. Every night the entire sampling area was surveyed initially to mark the identity of all the callers at their site of calling, and any non-callers that were found. The calling sites were marked using flags annotated with caller IDs and date of calling. Unmarked individuals were captured after the night’s observations, marked and released subsequently at their corresponding calling site. Further observations were initiated only after ensuring that all the callers had been located. Two types of sampling were used to collect data on male calling activity. A low-resolution scan sampling was used to collect calling activity data of all the callers on a given night. A high-resolution sampling was used for some individuals for validation of the population level low-resolution scans.

### Calling effort scans: low resolution

Two to three persons re-visited different marked calling sites every ten minutes and noted the activity of the callers without disturbing them. Calling behaviour was confirmed both visually and acoustically, from very close to the calling site. In rare cases when a caller stopped calling while being approached, the observer waited for a few seconds to re-confirm whether the male resumed calling behaviour. Activity of callers was noted using binary codes of zeroes, when males were not calling, and ones, when the males were calling. No observational scans were initiated after 2200 hours as calling activity reduces drastically beyond this time. Low-resolution scans were conducted on 31 consecutive nights, out of which on 19 nights, every individual was revisited at least ten times, thus covering a total of 100 minutes of calling activity at a resolution of ten minutes. On two out of 31 nights only seven scans (total of 70 minutes) were possible as it was very close to 2200 hours by the end of the last scan. 6–8 scans (60–80 minutes) were possible on five nights and 3–4 scans (30–40 minutes) on two nights due to heavy wind. Heavy wind affects calling behaviour of these animals and hence all observations were stopped when the wind speed exceeded 4 mph. On three out of 31 nights no scans were possible as wind speed picked up either at the time of or just after the initial survey. Callers were predominantly found in the core sampling area, though scans were also conducted in the buffer area to ensure larger spatial coverage. Individuals which started calling after the initial surveys were also located during the scans and included in subsequent scans.

### Calling effort scans: high resolution

Calling activity of some callers was surveyed every minute. The total duration of these high resolution scans per individual ranged from 60 to 126 minutes. High-resolution calling effort scans were conducted on 66 individuals in total. For 35 out of 66 individuals high-resolution scans were conducted on some of the nights during the 31- night consecutive sampling. For these 35 individuals both high-resolution and low-resolution scan data were obtained to enable a comparison between the calling effort estimates based on the two types of scans. For the remaining 31 individuals, calling songs and sound pressure levels were recorded along with high-resolution scans. For these 31 individuals, all the sound recordings and high-resolution scans were carried out in and around the sampling area which was used for low-resolution scans after the end of 31 consecutive nights of sampling. We ruled out any possible bias in the calling effort estimates for these 31 individuals which may arise due to sampling at a later period in the season, by comparing the distributions of calling effort estimates from these two sets (35 individuals during the season peak and 31 individuals towards the end of the season) and demonstrating no statistical difference between the two (*W* = 582, *P* = 0.88, Mann-Whitney U Test).

### Sound recording

Calling songs of individuals were recorded digitally and their SPL measured before initiating the high-resolution scans using a Brüel & Kjær ½″ microphone, Type 4189 (20 Hz to 20 kHz) and a Sound Level Meter, Type 2250 (Brüel & Kjær, Naerum, Denmark) with an in-built recording module at a distance of 20 cm in front of the calling male. High-resolution scans were initiated at least ten minutes after the sound recordings to ensure that the callers recovered from any disturbances during the song recording.

### Statistical analysis of temporal dynamics

All statistical analyses were carried out in the statistical programming language, R version 3.1.0 [55]. Only marked individuals were considered as data points for longitudinal (across-night) statistical analyses to avoid pseudo-replication. Individuals were considered for this analysis only if they were spotted at least once within that night. Therefore non-callers that could not be spotted within a night were not considered. Calling effort was defined as the proportion of times an individual was found calling out of the total number of times it was scanned. Using this definition, calling effort was separately estimated for both low-resolution and high-resolution scan data. We tested the concordance between the calling effort estimates based on high-resolution and low-resolution scans for 35 individuals for which both the data sets were available on the same night. A randomization simulation was also used where every 10^th^ data point was picked from the high-resolution calling effort data of a given caller, starting from the 1^st^ minute scan. This process was iterated with the starting point shifting by a minute till a minimum of seven scans would be possible within that iteration. The simulation thus generated a distribution of calling effort estimates based on scans every 10 minutes with varying total number of scans (13–7) depending on the total duration of the high-resolution scan for each of the 35 individuals. Finally the medians of these 35 simulated distributions were compared with the calling effort estimates based on the low-resolution scans using a non-parametric Mann Whitney-U test. We also compared the calling effort estimates of 35 individuals based on low-resolution and high-resolution scans directly using a paired-sample Wilcoxon signed rank test.

Within and among individual variation in calling effort across multiple nights was compared using repeatability analysis [56–58]. Calling effort estimates of 132 individuals across multiple nights based on low-resolution scans were used for repeatability analysis. Since calling effort was represented as proportions, we estimated repeatability in a generalized linear mixed model framework with a binomial error family and multiplicative overdispersion, in the package ‘rptR’ [59]. To investigate the effect of abiotic conditions within a night on within-individual variation in calling activity, co-variance in calling activity among males across multiple calling nights was analysed for 66 males that called on at least 5 nights. For each of these 66 males, Spearman correlation coefficient was estimated between the calling effort values of individuals on multiple calling nights and the median values of calling effort of the rest of the individuals within the same nights.

Trade-offs between within-night and across-night calling effort were investigated by determining the association between two calling effort parameters and the number of nights spent calling. The two calling effort parameters used for the correlational analysis were total calling effort and average daily calling effort. Total calling effort was calculated as the sum of the number of scans in which an individual called across all the nights spent calling of that individual. Average daily calling effort was estimated by dividing total calling effort with total number of scans summed across all the nights spent calling. In case of a trade-off between within- and across-night calling effort, average daily calling effort is expected to be negatively associated with the number of nights spent calling as those individuals that increase the number of calling nights will on average call less within a night. On the other hand, total calling effort may be expected to lack any association with number of nights spent calling if individuals calling on fewer nights and those calling on more nights have similar total calling effort across those nights. To determine the association between average daily calling effort and number of nights spent calling, we used generalized linear models with number of nights spent calling as the explanatory variable, and a binomial family of errors because average daily calling efforts were expressed as proportions. Number of nights spent calling can be spread over consecutive nights or more (in case of intermittent silent nights between the calling nights). Therefore, to control for the possible effect of the distribution pattern of the calling nights on the association between number of calling nights and calling effort, range of calling nights was included as a second predictor variable in this model. Range of calling nights was estimated as the number of nights between the first and last calling night of that individual. For individuals that called only on one night or on multiple consecutive nights, the range of calling nights and the number of nights spent calling were the same. However, in the maximal model we did not find any significant interaction between the two predictors, number of calling nights and range of calling nights (S1 Table). Even, excluding range of calling nights as a predictor did not lead to significant differences when compared using ANOVA (*P* = 0.11). Therefore, the final model included just the number of calling nights as the only predictor variable. The association between total calling effort and number of nights spent calling was determined by using generalized linear models with number of nights spent calling as the explanatory variable and a quasi-Poisson family of errors to account for over-dispersion in the data [60].

### Statistical analyses of spatial dynamics

Two reference points were chosen in the sampling area marked by flags. Distances of the calling sites, marked by different coloured flags, were measured from either of the reference flags using a meter tape. Angles subtended by the flags with respect to either of the reference points were measured using a survey precision compass (Survey Compass 17475780, error ±0.5°, conceptualized by Francis Barker and Sons Ltd., sold and serviced by Lawrence and Mayo, India) mounted on a tripod. These distances and angles were used to generate XY-coordinates for the individual calling sites across multiple nights. Using these Cartesian coordinates of the calling sites we quantified movement of individual callers across multiple nights. We estimated total absolute displacement as the linear distance between the calling sites of an individual’s first and last night of calling. Linear distances and angles were also measured between points representing an individual’s calling sites on successive nights spent calling. Individuals either called on consecutive nights or on nights interspersed with no signaling activity. Thus, to estimate successive-night displacement, these linear distances were divided by the number of nights between the two points on the successive nights spent calling. Individuals that called on more than two nights had more than one estimate of successive-night displacement. Thus, averages of successive-night displacements of an individual were estimated, using all the nights spent calling, and, separately, using only the consecutive nights spent calling. The distributions of average successive-night displacement calculated based on all the nights spent calling and on only consecutive nights were compared to study the effect of the variable intervals between the nights spent calling on across-night movement, using a Mann-Whitney U test. Angular movements of twenty-eight individuals that called on at least 8 nights, were used to test directionality of individual across-night movements separately, using Rayleigh’s test in the ‘circular’ package in R. To assess whether individuals moved towards the center of the aggregate or away from the aggregate, we estimated the distances of the calling sites from a central reference point in the sampling area for all the 28 individuals across their calling nights. If individuals progressively moved towards the center across multiple nights, then this distance should reduce generating a negative slope for the regression between distances from the center as a function of the number of nights. Similarly, a positive slope of the regression is expected if individuals moved away from the center. Therefore, a linear regression model was built with the distance from the center as the response variable, the number of nights as the continuous predictor and the individual ID as the categorical predictor.

### Trade-off between calling effort and calling song features

The digital recordings of the songs of 30 individuals were used for acoustic analysis. Temporal analyses were performed on 60 chirps from each of the recordings using customisable signal analysis programs (Chandra Sekhar Seelamantula, Electrical Engineering, Indian Institute of Science) in Matlab version 7 (Math-Works, Natick, MA, U.S.A.). The calling song of *Plebeiogryllus guttiventris* consists of chirps with varying number of syllables, ranging from two to six [32]. Chirp periods and chirp durations were therefore estimated separately for each of the different chirp types. The protocol of temporal pattern analysis and the features used were the same as in [51]. The five-syllable chirp is the most dominant chirp type [32] and thus appeared consistently in sufficient numbers in all the calling songs of the different individuals [51]. Thus five-syllable chirp period and duration along with the number of two-syllable chirps were used to investigate the correlation between calling effort and temporal features of the calling song. Correlation between calling song SPL and calling effort was also estimated. Since calling effort estimates were expressed in proportions, a Spearman’s rank correlation co-efficient was estimated for all the above correlations.

## Results

### Calling activity across multiple nights

**Fig 1:**
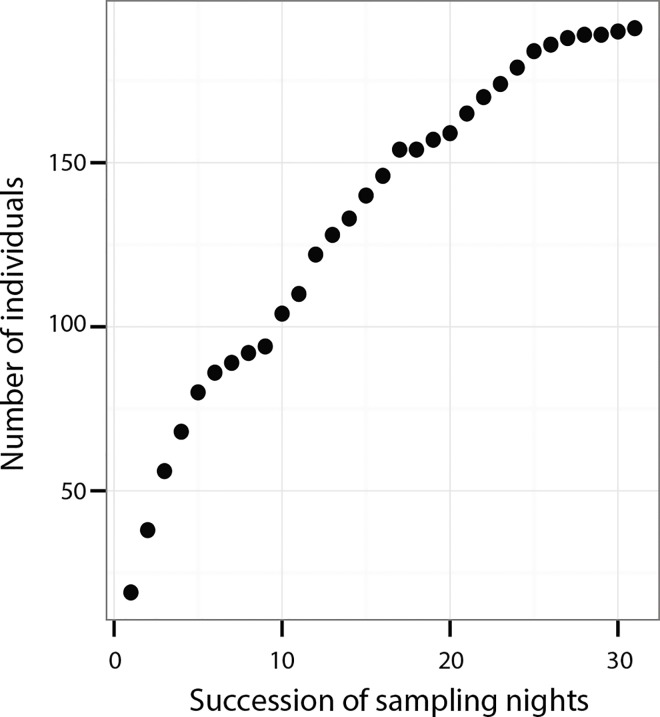
Encounter rates of new callers. An accumulation curve demonstrating the cumulative probability of encountering new callers over successive nights of sampling.

Out of a total of 286 individuals that were marked, 191 individuals (66.8%) called at least on one night. The rest of the 95 marked individuals were never seen again during the sampling. The total number of callers within a night ranged from 21 to 40 with a median of 32 (mean ± SD = 31.47±5.5). The number of new callers was the highest on the first few nights (Fig 1). The rate of finding new callers plateaued twice followed immediately by increments. The rate of finding new callers dropped considerably towards the end of the sampling period. The median number of new callers that joined the chorus was six per night.

### Validation of calling effort estimates based on low-resolution scans

**Fig 2:**
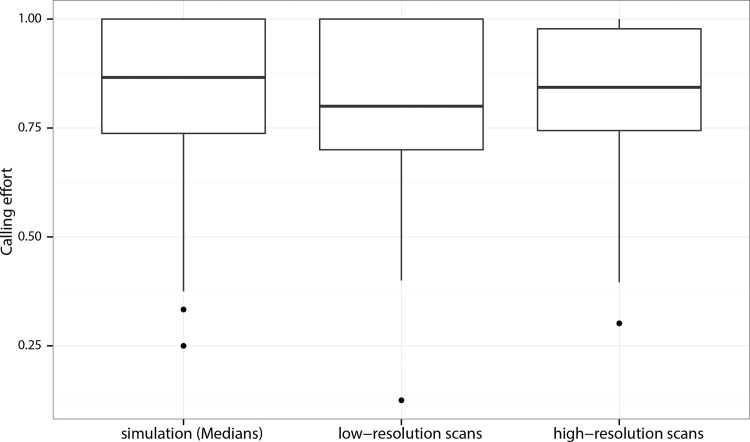
Validation of low-resolution scan-based calling effort estimates. Distributions of calling effort estimates based on low- and high-resolution scans and estimated medians generated by the simulation using high-resolution scan data. In the box and whisker plots, the central thick line depicts the median of the distribution while the box edges depict the 1^st^ and the 3^rd^ quartiles and the whiskers depict 1.5 times the interquartile range.

We did not find significant differences in the direct pairwise comparison of calling effort estimates based on the low-resolution and high-resolution scans of the same individuals (Fig 2, *V* = 182.5, *P* = 0.60). Moreover, the median calling effort when estimated from the simulation based on high-resolution scan data did not differ from the calling effort estimates based on low-resolution scans (Fig 2, *V* = 93, *P* = 0.7604). 75% of the pairwise differences between the simulated and low-resolution scan-based calling effort estimates were less than 0.14, with a median of 0.07, demonstrating the high concordance between the high-resolution and the low-resolution-based calling effort estimates (S1 Fig.).

### Are males consistent in their calling activity?

**Fig 3:**
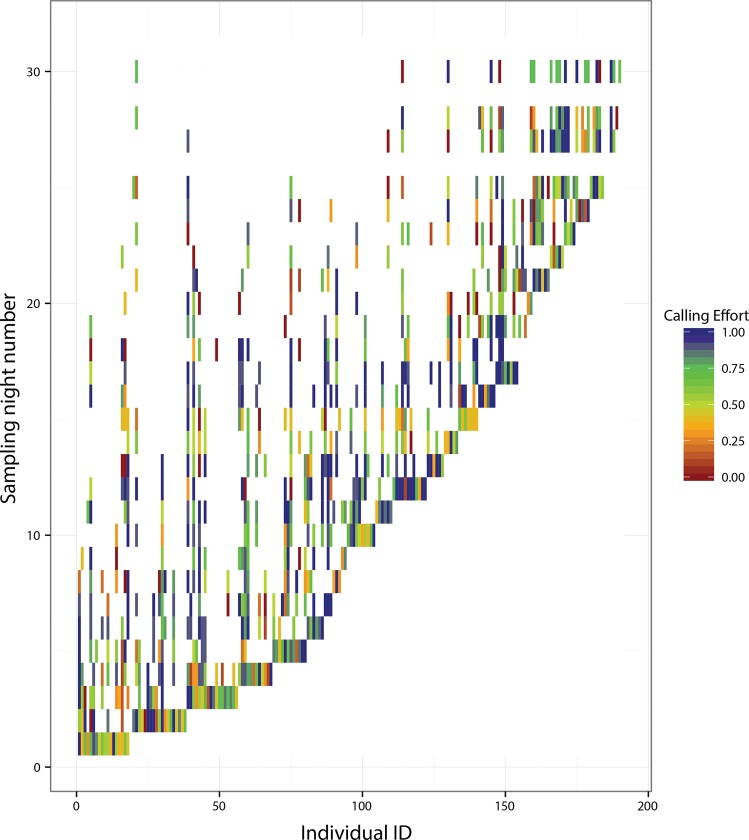
Within and among-individual variability in calling effort. Colour heat map depicting temporal variation in individual calling effort across 31 sampling nights. Red depicts low calling effort and blue depicts high calling effort.

Fifty-nine out of a total of 191 callers (30.8%) were observed to call only on one night and hence were not used for repeatability analysis. For the rest of the 132 callers, which called on at least two or more nights, the across-night repeatability of calling effort per night was 0.05±0.02 (mean ± SE). Callers had highly variable calling effort across nights, lacking any consistent pattern, which led to this low repeatability (Fig 3: difference in colour patterns along y-axis). Even within a night, callers seemed to vary in their calling effort (Fig 3: differences in colour patterns along x-axis). Callers did not, however, co-vary in their calling activity across multiple nights. For 66 callers tested, the median correlation co-efficient, between the individual’s calling effort estimates across multiple nights and the night-wise median of the rest of the callers, was 0.22 (S2 Fig.). The 1^st^ and the 3^rd^ quartiles of the distribution were -0.05 and 0.52 respectively indicating high variability in the correlation co-efficients (S2 Fig.).

**Fig 4:**
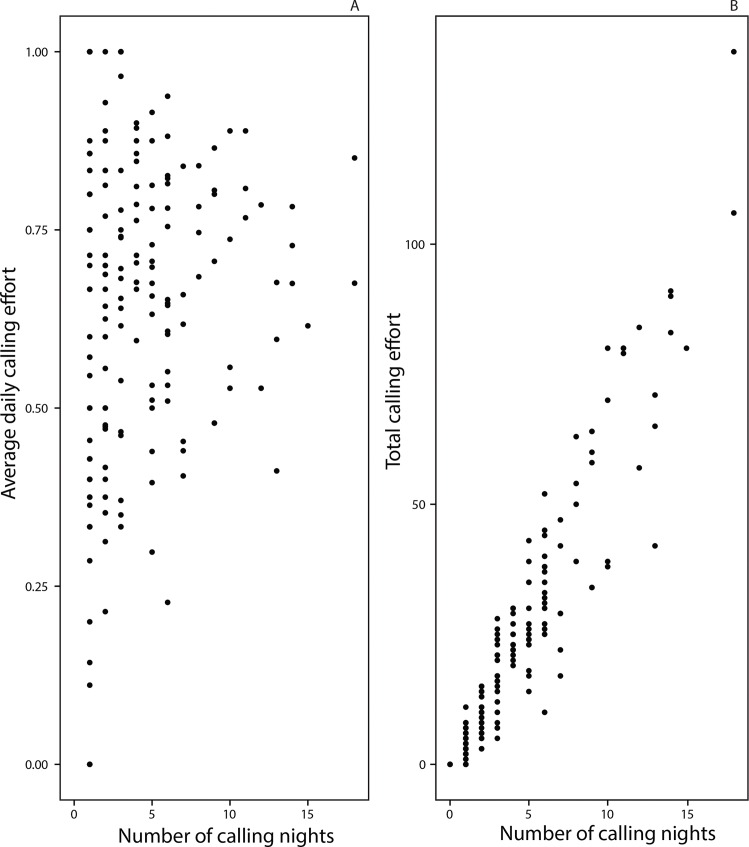
Association between calling effort and number of nights spent calling. (A) Average daily calling effort and (B) Total calling effort as a function of the number of nights spent calling. Total calling effort = Number of scans where an individuals was found calling across all the calling nights. Average daily calling effort = Total calling effort / Sum of scans per night across the calling nights

Average daily calling effort was not significantly associated with the number of nights spent calling (Fig 4A, *t* = 1.642, *P* = 0.0538). However, total calling effort increased with more nights spent calling (Fig 4B, *t* = 27.50, *P* < 0.001). 66% of the individuals called on less than 5 nights, with a median value of 3 nights (S3 Fig.).

### Across-night calling site fidelity

**Fig 5:**
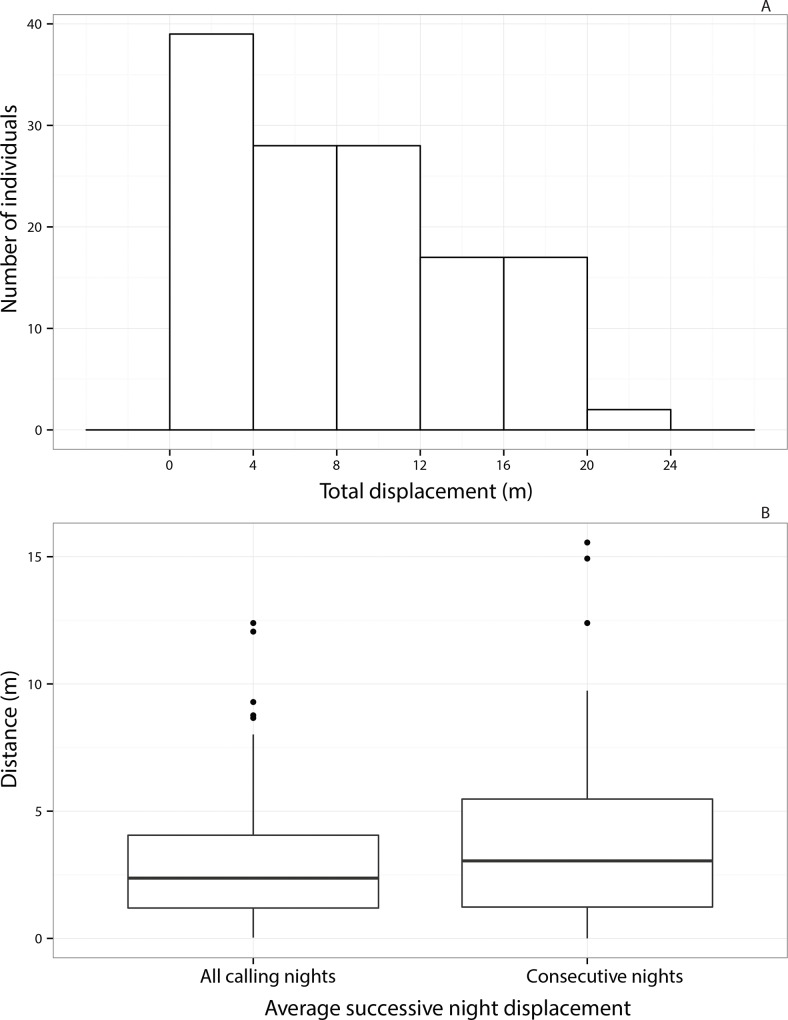
Across-night movement of callers. (A) Histogram showing distribution of total displacement of individuals and (B) Box and whisker plots of average successive-night displacement for pooled data (with intermittent silent nights) and for only consecutive nights. In the box and whisker plots, the central thick line depicts the median of the distribution while the box edges depict the 1^st^ and the 3^rd^ quartiles and the whiskers depict 1.5 times the interquartile range.

The median total displacement of callers was found to be 7.68 m with a range of .03 m to 23.78 m (Fig 5A). 75% of the callers moved less than 12.5 m between their first and last night of calling. Only 14 out of 191 callers (7.3%) showed total displacement of less than a meter, implying low across-night site fidelity for the population as a whole. The median value of average successive-night displacements was 2.36 m when calculated based on nights spent calling with variable numbers of intermittent silent nights. Moreover, the average successive-night displacements calculated using all the nights spent calling and only consecutive nights spent calling were not significantly different from each other (Fig 5B, *W* = 6096, *P* = 0.1067). Eighteen callers showed complete site fidelity (relative displacement = 0) across at least two successive nights spent calling, with only three out of 191 individuals maintaining their calling positions on three calling nights.

**Fig 6:**
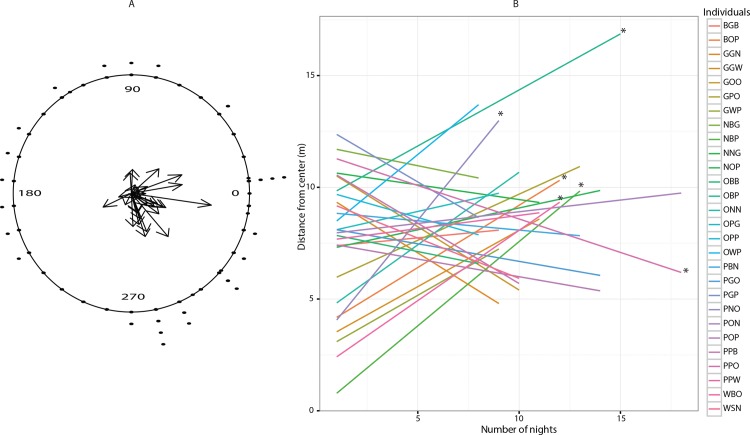
Directionality of across-night movement. A. Circular plots depicting directionality of across-night movement (as angles in degrees) along with their mean vectors for callers with at least 8 or more nights spent calling. B. Regression slopes of distances of the calling sites from the center of the aggregate across multiple nights against number of nights spent calling, for each of the 28 individuals that called on at least 8 nights. Trend lines corresponding to each individual are marked with different colours. The statistically significant regression slopes are marked with an asterisk.

Only one out of 28 callers showed significant directionality in its across-night movements (Fig 6A, S1 Table). The rest of the callers moved in random directions across different calling nights, showing an overall lack of directionality in across-night movements of individual callers (Fig 6A, S2 Table). For 6 out of 28 (21.4%) individuals, distance of the calling site from the center changed significantly over multiple nights (significant slopes, Fig 6B). Five out of these 6 individuals moved away from the center of the aggregate (significant positive slope, Fig 6B) while 1 moved towards the center (significant negative slope, Fig 6B).

### Trade-off between calling effort and calling song features

**Fig 7:**
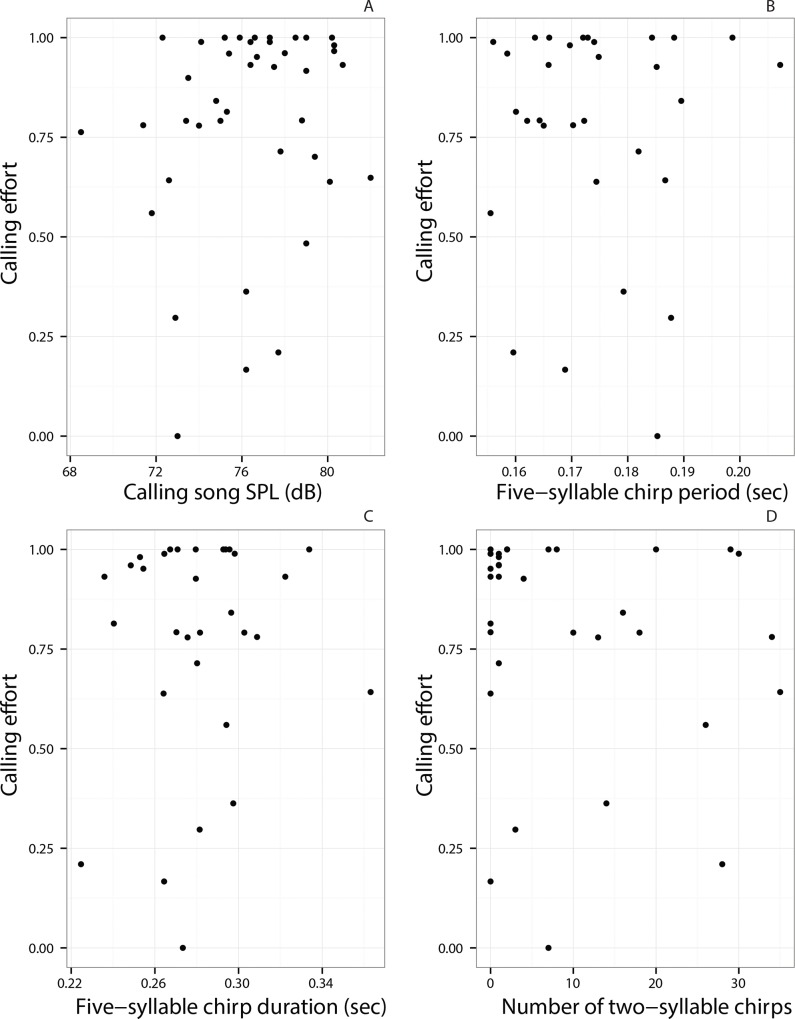
Trade-offs between calling effort and call features. Correlations between calling effort estimates based on high-resolution scans and (A) SPL (B) five-syllable chirp period (C) five-syllable chirp duration and (D) number of two-syllable chirps.

Calling effort of individuals was not significantly correlated with their SPLs (Fig 7A, Spearman’s *ρ* = 0.19, *P* = 0.26). Calling effort was also not correlated with temporal features of the calling song such as five-syllable chirp period (Fig 7B, Spearman’s *ρ* = 0.06, *P* = 0.76), five-syllable chirp duration (Fig 7C, Spearman’s *ρ* = 0.04, *P* = 0.83), and number of two-syllable chirps (Fig 7D, Spearman’s *ρ* = -0.11, *P* = 0.55).

## Discussion

### Within-individual variability in calling effort

We found across-night repeatability of calling effort to be low, implying that males are inconsistent in their calling effort across multiple nights. Observer-induced disturbances during scan sampling can cause inconsistent calling in crickets. In this study however, calling effort estimates based on the less invasive focal animal sampling (high-resolution scans) were concordant with those based on the potentially invasive scan sampling (low-resolution scans). Therefore, observer-induced disturbance is unlikely to have caused the inconsistency in calling activity seen in this study. Much of the variation in calling effort arises from the within-individual component of variance. Since repeatability sets the upper limit for heritability, a low value of .05 suggests a greater influence of environmental variables on male calling effort. A laboratory study on four species of field crickets found repeatability of calling effort to range between 0.43–0.96 [61]. In contrast, in anurans, a field study on the Gulf Coast toad, *Bufo valliceps*, estimated across-night repeatability of calling effort to range between 0.05–0.08 for two out of three years [62]. In this study, calling activity of males across multiple calling nights did not co-vary with other callers on the same nights. Hence, abiotic environmental factors, such as temperature, are less likely to explain the inconsistency in individual calling activity across multiple calling nights.

Calling effort in field crickets has been found to depend on the nutritional condition of the caller [20,24,21,63,23]. Thus lower calling effort repeatability as estimated in this field study could be due to the effect of nutrition, since the nutritional condition of a male need not remain constant over several nights. Moreover, field-based across-night repeatability estimates of calling song features which are affected by the immediate nutritional condition of males, such as chirp rate, have tended to be low in orthopterans as well as anurans [62,64–67]. The inconsistent calling effort of males across nights could be also due to acoustic interactions between callers and hence dependent on population densities. In a natural population of the field cricket species *Gryllus campestris L*, mating success of callers was found to be higher only under low population density [15]. Studies on other species of field crickets such as *Gryllus integer* and *Gryllus pennsylvanicus* have found significant selection gradients on male calling effort only under low population densities [18,19]. Thus at higher population densities, males can reduce their calling effort due to greater likelihood of female encounters thereby reducing the costs of calling [15]. However population densities are unlikely to fluctuate drastically within a span of a few days. Since most of the callers in this study called for a span of a few consecutive nights, population density fluctuations seem an improbable cause for the observed variation in male calling effort. Acoustic interactions between *P*. *guttiventris* callers could, however, be a factor leading to inconsistent calling activity. Due to high random movement between calling nights, males could also experience differential effects of female and predator densities if their spatial distributions are patchy and this may affect their calling behaviour.

### Relationship between calling effort and number of nights spent calling

In this study, average daily calling effort of individuals was not significantly associated with their number of nights spent calling. Total calling effort, on the other hand, increased with increasing number of nights spent calling. Increasing the number of calling nights at the cost of the amount of calling per night may give rise to a negative association between average daily calling effort and the number of nights spent calling. Such a trade-off may also lead to a lack of association between total calling effort and the number of nights spent calling. As individuals which call on more nights would on average call less within a night, total calling effort as a cumulative sum will remain similar to that of individuals which called on fewer nights in case of such a trade-off. In this study, the patterns of association between calling effort and number of nights spent calling, however, suggest that the amount of calling within a night does not affect the number of nights that male *P*. *guttiventris* call. Similarly, in another species of field cricket, *Teleogryllus commodus*, male life span did not affect average nightly calling effort and was positively associated with total calling effort, when studied using enclosures in their natural habitat [68].

### Trade-offs between calling effort and other song features

Calling effort was not correlated with any of the song features examined. The calling song features of *Plebeiogryllus guttiventris* males chosen for investigating trade-offs with calling effort are known to have diverse patterns of variability. In this species, across-night repeatability of calling song SPL was found to be high while that of chirp rate was low [51]. Both these calling song features are known to be energetically expensive in field crickets [1,27]. However, a lack of trade-off between either SPL or chirp rate, and calling effort, implies that *Plebeiogryllus guttiventris* males are not allocating their energy resources differentially to maximize one signal component at the cost of another. In *Gryllus texensis*, a trade-off between calling effort and call amplitude was observed only in males with consistently higher calling effort across several nights [42]. The inconsistency of calling activity across multiple calling nights in *P*. *guttiventris* males may affect such trade-offs. Even if males are consistent in their calling song SPL across multiple nights [51], a consistently louder male can have either high or low calling effort depending on its environment on that particular night. This environmental effect on calling effort can lead to a lack of correlation. On the other hand, a calling song feature such as chirp rate which is also affected by nutritional condition of the male, can be expected to trade-off with calling effort due to differential allocation of energy resources to either enhance the rate or effort of signaling. However, since callers were observed to call on few nights, the energetic demands of maintaining higher levels of calling effort and faster rate could be less severe leading to an overall lack of correlation.

### Low across-night calling site fidelity: implications for female mate sampling

Less than one percent of callers maintained their calling sites across multiple nights of calling. Lack of across-night site fidelity of calling sites implies no territoriality in these animals compared to other species of crickets such as *Scapteriscus acletus*, *Scapteriscus vicinus* and *Gryllus campestris* in which the males make burrows [53,69]. However, in a study on a different population of *Gryllus campestris L* across-night site fidelity was found to be very low with males occupying a burrow for an average of only one day [16]. The extent of across-night movement of *Plebeiogryllus guttiventris* callers was in fact similar to that found in this study [16]. Low across-night site fidelity was also observed in a haglid species, *Cyphoderris buckelli* [70] where the total displacement of callers was found to be similar to that in *P*. *guttiventris* males. High across-night movement of callers could also be an artifact of observer-induced disturbance during scan sampling. However, quantification of across-night movement among callers using non-invasive video monitoring techniques (as employed for *G*. *campestris* [4]) is not possible in a non-burrowing species such as *P*. *guttiventris* with low site fidelity and unpredictable movement trajectories. We did not, however, see any instances of observer-induced change in calling site within a night, making it unlikely that the across-nights movements were due to disturbance.

Low site fidelity among callers across successive calling nights can also affect the female mate sampling strategy by limiting the time window available for sampling. If females use strategies that require them to revisit potential mates (best-of-n or sequential comparison), then the visitations should ideally occur before a caller changes its calling site. Moreover, females using either a threshold or pooled-comparison mate sampling strategy [34,35,71] across multiple nights will have higher costs of sampling due to a greater likelihood of re-evaluating an already sampled male, if males change their calling site frequently across nights. Therefore in *P*. *guttiventris*, due to the low across-night site fidelity of callers, the time window of female mate sampling would be limited to within a night. Previous studies on different populations of *Plebeiogryllus guttiventris* as well as in other species of crickets have demonstrated high site fidelity of males during peak calling activity within a night [4,32,33,72].

### Implications for sexual selection on calling behaviour and calling song features

Calling effort in *P*. *guttiventris* was not associated with either calling song SPL or temporal features such as chirp rate or duration. This could imply that females may use these different components of the acoustic signaling behaviour as indicators of different male qualities. Since the ‘redundancy’ hypothesis predicts trade-offs between the different cues, the ‘multiple message’ hypothesis might be a more pertinent explanation for mate attraction based on different features of the calling behaviour [45].

Finally, the spatial and temporal axes of mate attraction (determined by the calling song SPL and calling effort respectively) seem to be uncoupled in this species. Thus individual males have the potential to maximize their mate attraction along one of the axes without being constrained by the other. Future studies on the determinants of variation in male calling effort and its association with mating success will be required to understand the role of sexual selection as a driver of signaling behaviour in this species.

## Supporting Information

S1 Fig Validation of low-resolution based calling effort estimates. Distribution of pairwise differences between the calling effort estimates based on the randomization simulation and those based on low-resolution scans (see text for details).
(EPS)

S2 Fig Among individual co-variance in calling activity across multiple nights. Distribution of Spearman correlation coefficients estimated between calling effort values of an individual across multiple nights and night-wise median calling effort of the rest of the callers within the same night.
(EPS)

S3 Fig Distribution of number nights spent calling. (EPS)

S1 Table Summaries of the 3 Generalized Linear Models. Column wise, 1^st^ model has two predictors and the interaction term, 2^nd^ model has just the two predictors without the interaction term and the 3^rd^ model has just one predictor. The model estimates (effect size) are given with the standard errors in the brackets below. Significance levels are indicated with asterisk.
(DOCX)

S2 Table Directionality of male across-night movement. Summary statistics and significance of Rayleigh’s test for each of the twenty-eight individuals that called on at least eight nights or more.
(DOCX)
