# Supplementary material for: Spatio-Temporal Dynamics of Field Cricket Calling Behaviour: Implications for Female Mate Search and Mate Choice
Source: PLoS One. 2016 Nov 7;11(11):e0165807. doi: 10.1371/journal.pone.0165807 (PMC5098824; doi:10.1371/journal.pone.0165807)
Supplement: S1 Table — Column wise, 1st model has two predictors and the interaction term, 2nd model has just the two predictors without the interaction term and the 3rd model has just one predictor. The model estimates (effect size) are given with the standard errors in the brackets below. Significance levels are indicated with asterisk. (DOCX) [file pone.0165807.s004.docx]

**S1 Table**: Summaries of the 3 Generalized Linear Models. Column wise, 1^st^ model has two predictors and the interaction term, 2^nd^ model has just the two predictors without the interaction term and the 3^rd^ model has just one predictor. The model estimates (effect size) are given with the standard errors in the brackets below. Significance levels are indicated with asterisk.

|  | | | |
| --- | --- | --- | --- |
|  | Dependent variable: | | |
|  |  | | |
|  | Average daily calling effort | | |
|  | (1) | (2) | (3) |
|  | | | |
| Calling Nights | 0.165^***^ | 0.091^**^ | 0.038 |
|  | (0.056) | (0.039) | (0.020) |
|  |  |  |  |
| Range of Calling nights | -0.019 | -0.036 |  |
|  | (0.024) | (0.023) |  |
|  |  |  |  |
| Calling Nights: Range | -0.005 |  |  |
|  | (0.003) |  |  |
|  |  |  |  |
| Intercepts | 0.282 | 0.473^***^ | 0.454^***^ |
|  | (0.149) | (0.108) | (0.108) |
|  |  |  |  |
|  | | | |
| Observations | 187 | 187 | 187 |
|  | | | |
| Note: | ^**^p<.05; ^***^p<0.01 | | |
