# Supplementary material for: Spatio-Temporal Dynamics of Field Cricket Calling Behaviour: Implications for Female Mate Search and Mate Choice
Source: PLoS One. 2016 Nov 7;11(11):e0165807. doi: 10.1371/journal.pone.0165807 (PMC5098824; doi:10.1371/journal.pone.0165807)
Supplement: S2 Table — Summary statistics and significance of Rayleigh’s test for each of the twenty-eight individuals that called on at least eight nights or more. (DOCX) [file pone.0165807.s005.docx]

**S2 Table. Directionality of male across-night movement**

Summary statistics and significance of Rayleigh’s test for each of the twenty-eight individuals that called on at least eight nights or more

| **S. no** | **Sample Size (n)** | **Mean Angle (degree)** | **Rho** | **P value** |
| --- | --- | --- | --- | --- |
| **1** | 7 | 351.700 | 0.68390 | 0.03143433 |
| **2** | 13 | 23.340 | 0.14730 | 0.76133922 |
| **3** | 12 | 272.800 | 0.27890 | 0.40173160 |
| **4** | 11 | 287.700 | 0.17300 | 0.72856348 |
| **5** | 10 | 339.100 | 0.23610 | 0.58447676 |
| **6** | 12 | 283.900 | 0.26940 | 0.42760721 |
| **7** | 11 | 323.600 | 0.28910 | 0.40810525 |
| **8** | 17 | 287.900 | 0.38120 | 0.08304314 |
| **9** | 17 | 289.400 | 0.22680 | 0.42330540 |
| **10** | 13 | 292.000 | 0.34630 | 0.21326449 |
| **11** | 9 | 337.100 | 0.29420 | 0.47162284 |
| **12** | 7 | 241.700 | 0.10770 | 0.92708438 |
| **13** | 10 | 332.900 | 0.09966 | 0.90968535 |
| **14** | 8 | 20.220 | 0.45560 | 0.19377164 |
| **15** | 13 | 205.700 | 0.26550 | 0.40789435 |
| **16** | 7 | 41.720 | 0.31210 | 0.52269973 |
| **17** | 14 | 41.470 | 0.07977 | 0.91752796 |
| **18** | 7 | 9.836 | 0.43670 | 0.27246611 |
| **19** | 9 | 195.400 | 0.11170 | 0.89902120 |
| **20** | 7 | 359.200 | 0.12180 | 0.90769319 |
| **21** | 8 | 87.250 | 0.20190 | 0.73413785 |
| **22** | 8 | 287.900 | 0.16390 | 0.81629549 |
| **23** | 8 | 337.800 | 0.31480 | 0.46692465 |
| **24** | 12 | 66.700 | 0.07870 | 0.93111464 |
| **25** | 9 | 310.500 | 0.47830 | 0.12723910 |
| **26** | 9 | 93.420 | 0.17270 | 0.77441180 |
| **27** | 8 | 322.100 | 0.16710 | 0.80970186 |
| **28** | 10 | 331.100 | 0.22050 | 0.62662066 |
